# Chemical pollution drives taxonomic and functional shifts in marine sediment microbiome, influencing benthic metazoans

**DOI:** 10.1093/ismeco/ycae141

**Published:** 2025-02-13

**Authors:** Lin-Lin Xu, Shelby E McIlroy, Yueqiong Ni, Isis Guibert, Jiarui Chen, Ulisses Rocha, David M Baker, Gianni Panagiotou

**Affiliations:** Microbiome Dynamics, Leibniz Institute for Natural Product Research and Infection Biology—Hans Knöll Institute, Adolf-Reichwein-Strasse 23, Jena, Thuringia, 07745, Germany; The Swire Institute of Marine Science, The University of Hong Kong, Cape D’Aguilar Road, Shek O, Hong Kong SAR, P.R. China; School of Biological Sciences, The University of Hong Kong, Pok Fu Lam, Hong Kong SAR, P.R. China; Simon F. S. Li Marine Science Laboratory, School of Life Sciences, The Chinese University of Hong Kong, Shatin, Hong Kong SAR, P.R. China; Microbiome Dynamics, Leibniz Institute for Natural Product Research and Infection Biology—Hans Knöll Institute, Adolf-Reichwein-Strasse 23, Jena, Thuringia, 07745, Germany; Cluster of Excellence Balance of the Microverse, Friedrich Schiller University Jena, Fürstengraben 1, Jena, Thuringia, 07743, Germany; Shanghai Key Laboratory of Diabetes Mellitus, Department of Endocrinology and Metabolism, Shanghai Diabetes Institute, Shanghai Clinical Center for Diabetes, Shanghai Sixth People’s Hospital Affiliated to Shanghai Jiao Tong University School of Medicine, 227 South Chongqing Road, Shanghai, 200025, P.R. China; The Swire Institute of Marine Science, The University of Hong Kong, Cape D’Aguilar Road, Shek O, Hong Kong SAR, P.R. China; School of Biological Sciences, The University of Hong Kong, Pok Fu Lam, Hong Kong SAR, P.R. China; Guangdong Provincial Key Laboratory of Food, Nutrition and Health, and Department of Nutrition, School of Public Health, Sun Yat-Sen University, Zhongshaner Rd 74, Guangdong, Guangzhou, 510080, P.R. China; Department of Applied Microbial Ecology, Helmholtz Centre for Environmental Research—UFZ GmbH, Permoserstrasse 15, Leipzig, Saxony, 04318, Germany; The Swire Institute of Marine Science, The University of Hong Kong, Cape D’Aguilar Road, Shek O, Hong Kong SAR, P.R. China; School of Biological Sciences, The University of Hong Kong, Pok Fu Lam, Hong Kong SAR, P.R. China; Microbiome Dynamics, Leibniz Institute for Natural Product Research and Infection Biology—Hans Knöll Institute, Adolf-Reichwein-Strasse 23, Jena, Thuringia, 07745, Germany; Cluster of Excellence Balance of the Microverse, Friedrich Schiller University Jena, Fürstengraben 1, Jena, Thuringia, 07743, Germany; Faculty of Biological Sciences, Friedrich Schiller University, Fürstengraben 1, Jena, Thuringia, 07743, Germany

**Keywords:** shotgun metagenomics, pollution, marine sediments, DNA metabarcoding, mediation analysis

## Abstract

Microbial communities in marine sediments contribute significantly to the overall health and resiliency of marine ecosystems. However, increased human disturbance undermines biodiversity and, hence, natural functionality provided by marine sediments. Here, through a deep shotgun metagenomics sequencing of the sediment microbiome and COI metabarcoding of benthic metazoans, we demonstrate that >50% of the microorganisms’ and metazoan’s taxonomic variation can be explained by specific chemical pollution indices. Interestingly, there was a significant correlation between the similarity in microbiome communities’ taxonomical and functional attributes and the similarity of benthic metazoans community composition. Furthermore, mediation analysis was conducted to evaluate the microbiome-mediated indirect effect, suggesting that microbial species and functions accounted for 36% and 26%, respectively, of the total effect of pollution on the benthic metazoans. Our study introduces a multi-level perspective for future studies in urbanized coastal areas to explore marine ecosystems, revealing the impact of pollution stress on microbiome communities and their critical biogeochemical functions, which in turn may influence macrofaunal composition.

## Introduction

Coastal marine habitats, including a large variety of habitats such as estuaries, mangroves, and coral reefs, are the world’s most heavily used and commercially important ecosystems [1]. These ecosystems provide numerous benefits, including food, recreation, protection from storms and erosion, and economic opportunities. For example, marine fisheries provide >260 million livelihoods, generating sizeable revenues for many countries, including US$ 80 billion dollars in export revenues for developing countries [2]. Despite the important services that coastal marine ecosystems bring to humanity, they face various anthropogenic threats [1]. Particularly, heavy metal pollution in coastal marine environments from domestic and industrial effluents has drawn attention for decades [3] and continues to be a focal point, especially in developing countries [4, 5]. Additionally, polycyclic aromatic hydrocarbons (PAHs), which exist as hazardous complex mixtures in nature and accumulate in sediment environments, pose potential risks to benthic organisms [6] and human health [7].

Sedimentary habitats span the majority of the ocean floor and make up more than two-thirds of the Earth’s surface, support diverse benthic communities. Microbial communities in marine sediments play an important role in biogeochemical cycling [8–10], and provide chemical cues that are essential for the recruitment of marine invertebrates [11]. Urban development has increased drastically in the last few decades, bringing pollution and impacting the benthic marine ecosystem, including marine sediment microbiome and benthic metazoans. Pollutants have been demonstrated to affect both composition and function of sediment microbial communities, including nitrogen cycling [12] and antibiotic resistance genes (ARGs) [13]. Furthermore, nematodes, benthic metazoans commonly found in marine sediments, exhibit bioaccumulation of heavy metals, which can lead to biomagnification and increase the transfer of contaminants along food webs to larger organisms [14]. Evidence indicates that human-introduced antibiotics alter environmental microbiomes, shifting zooplankton microbiomes and affecting their growth [15]. Additionally, microbial biomass likely serves as a crucial food source for hadal meiofauna, driving increased abundance and variations in their densities within sediments [16]. However, while changes resulting from pollution stressors on marine sedimental microbial diversity and function are well established, their impact on macrofauna has remained largely unexplored. Due to our limited understanding of the essential microbial functions in the marine ecosystem, our capacity to benefit from such interactions in conservation practices is severely constrained.

Marine animals live in close relationships with microorganisms that modulate the environmental conditions animals are exposed to, impacting the resilience and adaptation of marine holobionts [17]. The extent to which the marine sediment microbiome taxa and function may affect macrofauna, however, has not been thoroughly investigated. Here, we examined the role of the marine microbiome in the coastal maritime zones across various pollution sources by integrating sediment quality indices, marine sediment microbiomes, and benthic metazoans. To assess benthic metazoan communities, we used Autonomous Reef Monitoring Structures, ARMS, well-established, standardized, passive samplers which, when combined with metabarcoding approaches, are ideal to characterize the largest portions of marine biodiversity [18, 19]. Deep metagenomics sequencing of the marine microbiomes allowed the construction of high-quality assemblies for functional characterization of the microbiota and revealed the shifts triggered by pollution stressors. By combining a variety of methodological approaches, we demonstrated a strong association between pollution-driven microbiome shifts and benthic metazoans. The identified microbiome-benthic metazoan signatures not only further characterized the coastal marine ecosystem under pollution stress, but also underscored the necessity for more holistic approaches in conservation practices. The comprehensive understanding of marine ecosystems from multiple perspectives will shed light on protecting marine biodiversity, ecosystem services, and the sustainable use of marine resources in the context of a rapidly developing metropolis’s coastal shorelines.

## Materials and methods

### Sampling sites and sample collection

Seven locations [San Shek Wan (SSW), Peng Chau (PC), Center Island (CI), Sham Wan (SW), Cape D’aguilar (CDA), Bluff Island (BI), Tung Ping Chau (TPC)] in Hong Kong’s major coastal marine were studied. ARMS were deployed near the shore at ~4 meters depth in Jan 2018 (*n* = 3 per site) and July 2018 (*n* = 3 per site), and retrieved 12 months later in Jan 2019 and July 2019, respectively. The three ARMS were placed ~5 meters apart at each site. Just prior to the retrieval of each ARMS, 50 g of sediments from the top 10 cm of seafloor, ~10 cm from ARMS was scooped into a sterile 50 mL falcon tube. Sediments were snap frozen in liquid nitrogen once on board and stored at −80°C upon return to the laboratory for subsequent metagenomic analyses.

### Pollution data and benthic metazoans data collection

Geochemical indicators across in year 2019 were obtained from the Hong Kong Environmental Protection Department’s periodic sediment monitoring database (https://cd.epic.epd.gov.hk/EPICRIVER/marine/?lang=en). Monitoring stations were selected based on their proximity to the ARMS deployments and only the nearest date to sediment collection was considered. The average distance between the pollution monitoring site and the sampling locations across all seven sites is 4.4 kilometers (Table S1). The details of ARMS processing, metazoans data collection and processing are available in McIlroy et al [20]. Briefly, each ARMS is a ~ 22 × 22 × 25 cm structure consisting of 9 stacked polyvinyl chloride (PVC) plates with alternating open and partially closed spaces between. Following field deployment, during which small motile and sessile organisms naturally recruit and settle onto ARMS, the ARMS are removed and taken apart. All motile organisms between 100 and 500 um are collected by sieving, and the bulk sessile communities are scrapped from the plate and homogenized. Those samples were preserved in 90% EtOH, extracted using Qiagen Power Soil kits, and COI sequences are amplified and sequenced using standard metabarcoding techniques [21]. The forward and reverse sequence reads were processed with Cutadapt [22] and DADA2 pipeline [23] for quality control. Sequences were further aligned to Moorea BIOCODE library with MASCE [24]. Only sequences that had zero stop codons, zero frameshifts, zero insertions and no more than three deletions were retained and the remaining amplicon sequencing variants (ASVs) were clustered using VSEARCH [25] into operational taxonomic units (OTUs) based on 97% sequence similarity. OTUs were assigned to taxonomic groups using a custom-built database including the CO-ARBitrator database [26], sequences from GEOME [27] and 735 barcodes generated from large motile macrofauna of the same ARMs using VSEARCH within QIIME2 [28]. A 95% sequence similarity threshold was set for species-level taxonomic assignments where feasible. Unassigned OTUs underwent a second round of identification with an 80% threshold, followed by BLAST searches against the entire National Center for Biotechnology Information (NCBI) NT database (as of May 2021; word size = 7; max e-value = 5e-13). Taxonomy is assigned based on the lowest common ancestor (LCA) of the top 100 hits.

### Deoxyribonucleic acid extraction and metagenomic sequencing

Deoxyribonucleic acid (DNA) from the sediment samples was extracted using the Mag-Bind Stool DNA 96 Kit following the manufacturer’s instructions. DNA concentrations were verified using the Qubit Fluorometer and DNA quality was assessed via agarose gel electrophoresis (using Takara λ-Hind III digest and the Tiangen D2000 marker) prior to library construction. DNA libraries were constructed using the BGI in-house library preparation kit. Metagenomic shotgun sequencing was then performed on an Illumina HiSeq Xten (150 bp PE) platform.

### Taxonomic profiling

Human contamination reads were removed after mapping raw sequencing paired-end reads to human genome sequences with bwa mem using default parameters. After that, we used pipelineForQC.pl [29] to perform quality control and remove adapter regions, low-quality reads, and duplicate reads. DIAMOND v2.0.14.152 BLASTX [30] was used to map these reads to the NCBI nonredundant (nr) database (as of February 2024) using an e-value <1e−10 cutoff. The LCA algorithm was used to estimate the taxonomic composition of the samples using the daa-meganizer from MEGAN6 [31] with default parameters (blast hits with a bit score <50, and hits outside the top 10% of the highest bit score were excluded). Reads with bacteria or archaea information were used for further analyses. Prokaryotic community profiles were constructed at each taxonomic level for further statistical analysis.

### Microbial community composition

R package vegan [32] was used to compute alpha diversity indices describing each sample’s microbial community composition. The Shannon and Simpson indices were used to assess alpha diversity based on the relative abundance. Bray–Curtis distances calculated by R package vegan based on the relative abundance of each taxon at different levels were used to estimate community dissimilarities. Adonis from the vegan package in R was used as a permutational multivariate analysis of variance (PERMANOVA) test to determine the significance of a variable in determining distance variation (the number of permutations were set as 999). Microbiome vectors were overlaid onto the PcoA using the envfit function within the vegan package and the false discovery rate (FDR) is adjusted by Benjamini–Hochberg’s (BH) multiple-testing correction method (*P* < .05, FDR < 0.1). Distance-based redundancy analyses (dbRDA) were performed with R package vegan, using Bray–Curtis distances. dbRDA analyses were first performed using the capscale function from vegan with 35 pollution indices collected from sediment. A nonredundant contribution to variation of these variables was calculated using forward stepwise variable selection via the ordiR2step function from vegan. Univariate analyses with both chemical pollution indices and six environmental variables were calculated and model p values were corrected using BH multiple-testing correction to select variables with BH-adjusted *P* values <.05. Hierarchical clustering of pollution indices, selected through stepwise dbRDA, was performed using the ComplexHeatmap package (v2.6.2) in R. The clustering was based on the Ward.D method with Euclidean distance.

### Metagenome data assembly and functional annotation

Clean reads passed quality control were used for assembly by metaSPAdes from metaWRAP v1.2.3 [33]. The resulting assemblies were subjected to eukaryotic contig assignment using EukRep v.0.6.7 [34]. Eukaryotic contigs were filtered out from the original assemblies, resulting in individual “prokaryotic-enriched” assembly files for each sample. Metagenemark v3.38 [35] was used to predict genes from each assembled genome. Predicted genes from each assemblies were aligned to the database of Clusters of Orthologous Genes (COG) [36] and the KEGG Orthology (KO) database [37] built by KOBAS v3.0 [38] using DIAMOND [30] v0.9.19.120 BLASTP (−e 1e−10, best hits reserved). The identified COGs were further annotated into different pathways and categories based on predefined collections in the COG database and were quantified by copies per million (CPM). The identified KOs were quantified by CPM and a curated heavy metal resistance KO list [39] based on BacMet database [40] were used to retrieve metal resistance KOs.

### Data analyses and statistics

#### Differential analyses

Differential abundance analyses of prokaryotic- and benthic metazoans community profiles were performed using R package MaAsLin2 [41] for prokaryotic taxa profile (compound Poisson linear model: CPLM model), prokaryotic function profile (linear model: LM model; log transform), and benthic metazoans’ taxa profile (linear model: LM model) to identify pollution-associated microbes (season as fixed effect) and season-associated microbes (sampling sites as random effect).

#### Correlation analyses

Inter-dependencies among the chemical pollution indices and environmental variables were evaluated using Pearson correlation with R package Hmisc. Correlations between pollution-associated microbes and chemical pollution variables were calculated using partial Spearman correlation analysis, adjusted for season, with the R package ppcor. Correlations between heavy metals and heavy metal resistance KOs markers were assessed using Spearman correlation with the R package ggpubr.

#### Richness comparison

Pearson correlation between community richness was calculated by R package ggpubr. Mantel test with function mantel from R package vegan, using Spearman’s correlation coefficient with 1000 times permutations was used to analyse the associations of dissimilarity between microbiome community and benthic metazoans community. Bray–Curtis dissimilarity matrices for the microbiome community and Jaccard dissimilarity matrix for the benthic metazoans community were computed to perform this test.

#### Mediation analyses

Mediation associations on the community level were evaluated by R package method for multivariate omnibus distance mediation analysis (MODIMA) [42] with Euclidean distance for chemical pollution indices, Bray–Curtis distance for the microbiome community and Jaccard distance for the benthic metazoans. R package LDM-med [43] is used to validate the mediation associations on the community level. Individual-level mediation analysis was carried out with the mediate function from the R package mediation (version 4.5.0) [44], using pollution-associated features identified with R package MaAsLin2 (*P* ≤ .05, FDR ≤ 0.2), including microbial species, COG pathways, and benthic metazoans OTUs. The BH procedure was applied to control FDR.

## Results

### Specific pollutants explain microbiome community variation in marine sediments

**Figure 1:**
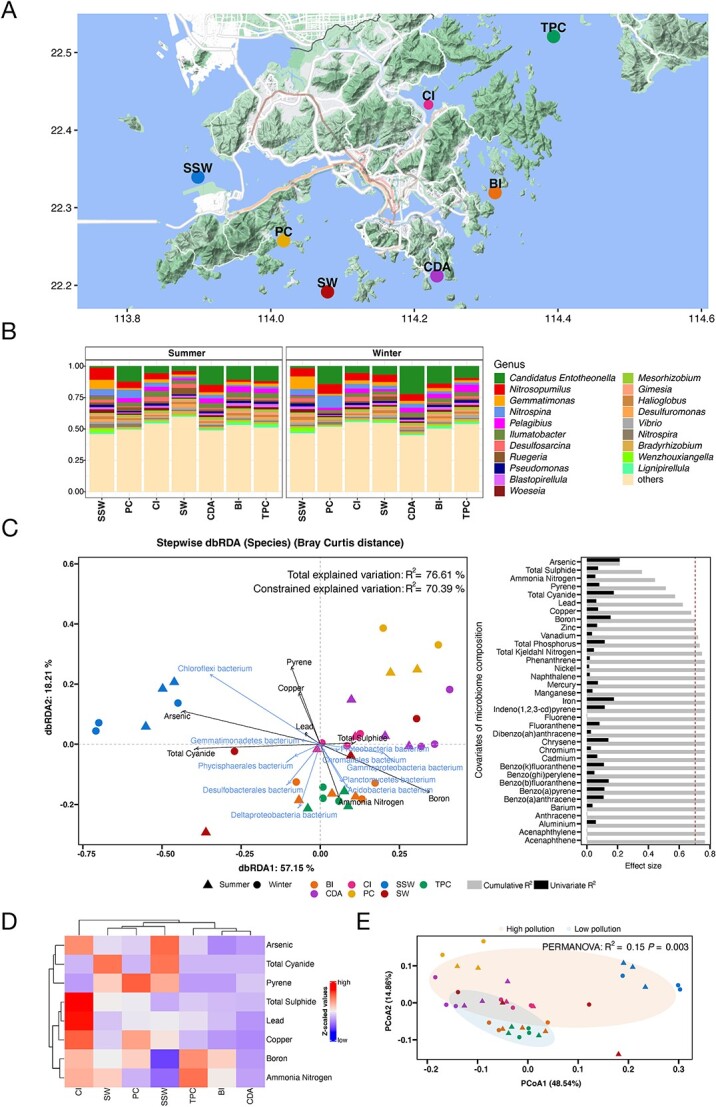
Sedimental pollution shapes microbiome community. (A) Map of seven sampling sites in Hong Kong: SSW, PC, SW, CDA, CI, BI, and TPC. Map of the geographic distribution of the samples were constructed using the R package ggmap. (B) Taxonomic profile of the prokaryote community at the genus level. (C) dbRDA plot based on Bray–Curtis distance. The red dashed line represents the cut-off for significant nonredundant contribution to the multivariate model. (D) Heatmap of microbiome associated pollution variables in seven sites with hierarchical clustering. Averaged and scaled value of pollution variables are used as heatmap colors reflecting standard deviations from the mean. Clustering is based on Euclidean distances. (E) Beta diversity of prokaryote community at the species level using Bray–Curtis distance.

Seven sites across Hong Kong water zone (Fig. 1A) chosen for this study were sampled in 2 seasons (2019: summer and winter, *n* = 37). Thirty-five sediment related pollution indices and six environmental variables were gathered from the monitoring stations close to each sampling site’s depth and geographical location (Table S1). To characterize the sediment microbiome community, deep metagenomics sequencing (to facilitate assembly in the downstream analysis of the microbiomes) of the sediment samples generated on average 70 million reads (standard deviation: 10 million reads) of ~150 nucleotide length per sample after quality control (Table S2). To reveal the composition of the marine sediment prokaryotic communities, we extracted bacterial and archaeal reads among samples at different taxonomic levels from kingdom to species (Table S3). The relative abundance of bacteria and archaea in the sediments was 97.5% and 2.5%, respectively. At the phylum level, the most abundant bacteria and archaea were *Proteobacteria* (59.0 ± 5.9%), *Planctomycetes* (9.9 ± 2.1%), *Chloroflexi* (5.2 ± 4.4%), *Bacteroidetes* (5.1 ± 2.1%), and *Acidobacteria* (3.6 ± 0.7%). The most abundant genus and species were *Candidatus Entotheonella* (9.8 ± 7.0%) and *Gammaproteobacteria bacterium* (13.3 ± 2.4%; Fig. 1B; Fig. S1).

We explored potential associations between the species-level microbiota composition and pollution indices. More than 50% of the variance in the species-level microbiota composition was significantly explained by all covariates together (dbRDA, R^2^ = 76.6%, *P* = .001). In univariate analysis, 11 of these covariates showed a significant correlation to microbiota composition (dbRDA, FDR < 0.05). These significant covariates were classified as heavy metal, nutrient, PAHs, and inorganic pollutants (Table S4). Considering the potential multicollinearity between covariates (Fig. S1), we further applied stepwise dbRDA to identify the group of pollution indices that could explain variation in the microbiome community without redundancy. Out of all covariates, arsenic, total sulfide, ammonia nitrogen, pyrene, total cyanide, lead, copper, and boron were found to explain both significant and nonredundant fractions of compositional microbiome variation (Fig. 1C, stepwise dbRDA, R^2^ = 70.4%, *P* = .001). Environmental variables were assessed; however, none showed significant contributions in the univariate dbRDA analysis, nor were they selected in the stepwise dbRDA model (Table S4). These microbiome community related pollution indices selected by stepwise dbRDA were further used for the hierarchical clustering of the seven sites revealing complex and diverse pollution sources for each site (Fig. 1D). Sampling sites (−TPC, BI, and CDA−) were clustered together showing relatively low pollution level, whereas sampling sites (−SSW, PC, and CI, and SW−) showing the opposite. SSW, PC, and CI are significantly impacted by human activities [45, 46], leading to high nutrient loads and pollution.

To evaluate the differences in community diversity between high- and low-pollution sites, as clustered in Fig. 1D, we calculated the alpha- (Shannon and Simpson diversity) and beta-diversity. Beta diversity (Bray–Curtis distance) highlighted significantly different microbiome communities among the high and low pollution sites at the species and genus (Fig. 1E; Fig. S2) level (PERMANOVA test, species: R^2^ = 0.15, *P* = .003, genus: R^2^ = 0.18, *P* = .001). However, no significant difference between seasons (summer and winter) were observed in beta-diversity (PERMANOVA test: *P* > .05) or alpha-diversity (Wilcoxon signed-rank test: *P* > .05; Fig. S2).

### Specific pollutants associated with abundance changes of bacteria taxa in marine sediments

**Figure 2:**
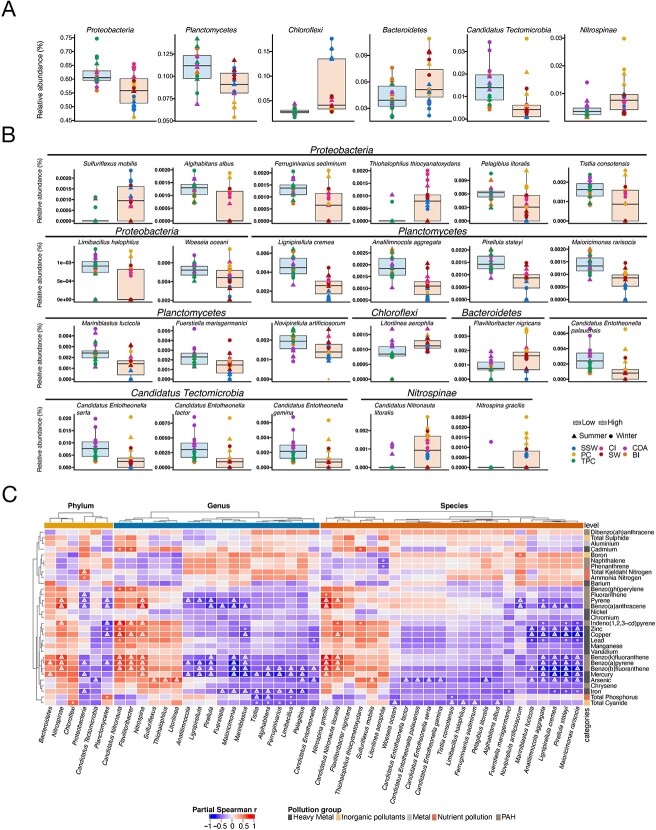
Microbial abundance changes related to pollution. (A) Significantly differentially abundant microbial phyla between low- and high-pollution sites identified by R package MaAsLin2 (*P* ≤ .05, FDR ≤ 0.2). (B) Significantly differentially abundant microbial species between low- and high-pollution sites identified by R package MaAsLin2 (*P* ≤ .05, FDR ≤ 0.2). (C) Partial Spearman correlations (adjusted for the season) between pollution-associated microbes and environmental pollution variables. Correlations with raw *P*-value <.05 were labeled with a circle. Correlations with raw *P*-value <.05 and BH *P*-value <.2 were labeled with a triangle.

Since our sampling included both the summer and winter periods, we built linear mixed-effects models with sampling sites as a random factor to find significant changes in the abundance of bacteria and archaea associated with season (Fig. S3). In total, we found 3 genera and 14 species significantly affected by the season using CPLM model implemented in MaAsLin2 (*P* ≤ .05, FDR ≤ 0.2), in which *Ilumatobacter* and *Synechococcus* are more abundant in summer, whereas *Candidatus Promineofilum* is enriched in winter. Therefore, when we subsequently explored the microbiome relative abundance changes at different taxonomic levels between high- and low- pollution sites, we adjusted for the season using CPLM model implemented in MaAsLin2 (*P* ≤ .05, FDR ≤ 0.2). We found 19 phyla, 95 genera and 93 species were significantly affected by the pollution (Fig. 2A and B; Fig. S3; Table S5). Six of the nineteen phyla have the most species (number > 3) significantly affected by pollution, including both pollution-sensitive phyla (*Proteobacteria*, *Planctomycetes*, and *Candidatus Tectomicrobia*) and pollution-tolerant phyla (*Chloroflexi*, *Bacteroidetes*, and *Nitrospinae*). Consistent with previous studies [13] the phylum *Planctomycetes* showed a significant decreasing abundance from low to high-polluted sites (Fig. 2A), which our data suggest is season-independent. In contrast, the phylum *Nitrospinae*, including *Nitrospina* and *Candidatus Nitronauta*, showed an opposite trend (Fig. 2A). Although *Nitrospinae* is important to the nitrogen cycle in marine ecosystems, it can also contribute to the eutrophication [47]. In addition, we found a decreased abundance of genus *Ilumatobacter* and its species *Ilumatobacter nonamiensis* and *Ilumatobacter coccineus* associated with the elevated pollution level (Table S5), which may be related to the sensitivity of those microorganisms to metal contamination [48]. *Proteobacteria*, known for driving carbon cycling through various processes, such as primary production (sulfur and methane oxidation [49]), heterotrophy [50], and photosynthesis [51], was found in our study to include both pollution sensitive and pollution tolerant species (Fig. 2B).

To delineate the associations between pollution concentrations and microbial abundances in marine sediments we applied partial Spearman correlation analysis (*P* < .05, FDR < 0.2, adjusted for the season; Fig. 2C). From the significantly affected by the pollution taxa, our analysis showed that *Planctomycetes* (including genus—*Pirellula*, *Mariniblastus*, and *Maioricimonas*—and species—*Pirellula staleyi*, *Mariniblastus fucicola*, and *Lignipirellula cremea*) was significantly negatively correlated with heavy metals and PAHs. In addition, *Nitrospinae* (including genus *Nitrospina* and species *Nitrospina gracilis*) was significantly positively correlated with PAHs. In addition, we observed *Ilumatobacter* and more specifically *Ilumatobacter coccineus* to be significantly negatively correlated with heavy metal (arsenic and iron) and PAHs pollution indices (Fig. S4).

### A functional adaptation of the marine microbiome to pollution-associated stress

**Figure 3:**
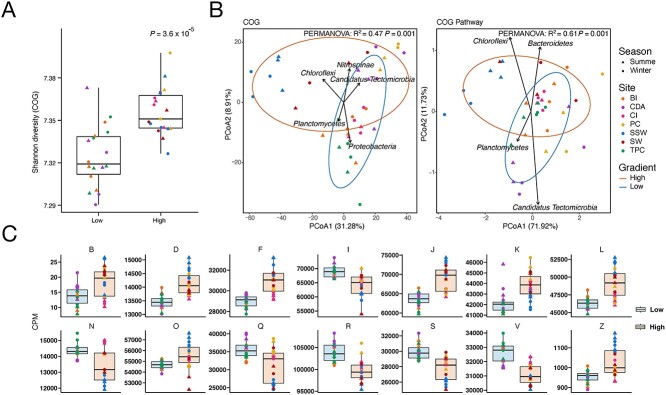
Microbial functional adaption to pollution stress. (A) Comparison of Shannon diversity based on COG terms between low- and high-pollution sites. (B) PCoA plot based on COG terms and COG pathways using Euclidean distance. Confidence ellipses: 95% confidence level. Individual lines with an arrow show vector matrices of the significant pollution-related microbial taxa. (C) Significantly changed COG categories between low- and high-pollution sites identified by R package MaAsLin2 (*P* ≤ .05, FDR ≤ 0.2). B, chromatin structure and dynamics; D, cell cycle control, cell division, chromosome partitioning; F, nucleotide transport and metabolism; I, lipid transport and metabolism; J, translation, ribosomal structure, and biogenesis; K, transcription; L, replication, recombination, and repair; N, cell motility; O, posttranslational modification, protein turnover, chaperones; Q, secondary metabolites biosynthesis, transport and catabolism; R, general function prediction only; S, function unknown; V, defense mechanisms; Z, cytoskeleton.

Following quality control, ~2.6 billion filtered reads were used to assemble contigs for each individual sample, resulting in an average of 2.7 billion bp in assembled metagenome (average N50: 261 bp) per sample (Table S6). After removing eukaryotic contigs (EukRep), we predicted genes on constructed prokaryote-enriched metagenome assemblies for functional comparisons. A total of 4646 COG terms, ranging from 4227 to 4415 in each sampling site, were identified and categorized into 63 COG pathways. Shannon diversity index was used to calculate the alpha diversity of COGs. Significant differences between pollution sites were found in alpha diversity based on COG terms (Wilcoxon test, *P* < .05, Fig. 3A).

Beta diversity calculated by Euclidean distance of COG terms and COG pathways revealed significantly different functional profiles between high and low pollution sites (PERMANOVA test, *P* < .05, Fig. 3B). We did not observe any significant seasonal differences in alpha or beta diversity in COG terms and COG pathways (Wilcoxon signed-rank test: *P* > .05, PERMANOVA test: *P* > .05). The overall difference in COG terms across pollution sites appears to be significantly driven by different microbes (envfit from R package vegan, *P* < .05, FDR < 0.05, Fig. 3B), the abundance of which varies significantly depending on the pollution. Phylum *Planctomycetes* and *Proteobacteria* were significantly associated with the functional signature of the microbiome in the low pollution assemblage, whereas phylum *Nitrospinae* and *Chloroflexi* were associated with high pollution assemblages.

To identify the functional categories of the prokaryotic communities mostly affected by the pollution, we examined the COG categories, COG terms and pathways that showed overall significant differences by R package MaAsLin2 (*P* ≤ .05, FDR ≤ 0.2). In total 14 COG categories (Fig. 3C) showed significant differences, suggesting a substantial influence of pollution on functional profiles of the microbiome in marine sediments. Significantly more genes related to “Replication, recombination and repair (L)” were found in the high pollution sites as previously shown in heavy-metal contaminated sediments [52]. We also observed significantly more genes related to “Translation, ribosomal structure and biogenesis (J)” in high pollution sites. The relative abundance of 10 COG pathways and 751 COG terms was significantly reduced as the pollution increased, while 29 COG pathways and 1126 COG terms had an opposite trend (Fig. S5 and Table S7). Several metabolic pathways, including fatty acid biosynthesis, biotin biosynthesis, asparagine biosynthesis, and serine biosynthesis were found to decrease at high-polluted sites. Most of the increased pathways are related to COG categories J (translation, ribosomal structure and biogenesis), E (amino acid transport and metabolism), and F (nucleotide transport and metabolism). In highly polluted areas, we discovered an increase in genes related to FOF1-type ATP synthase, which is important for the synthesis of ATP [53]. To assess whether heavy metals specifically influence microbiome function, we examined a set of marker KOs for metal resistance mechanisms [39, 40]. We found that several metal resistance KO markers were significantly positively associated with their corresponding heavy metals, such as arsenic, cadmium, copper, lead, and zinc (Fig. S5).

### Sessile benthic metazoan structure affected by pollution

**Figure 4:**
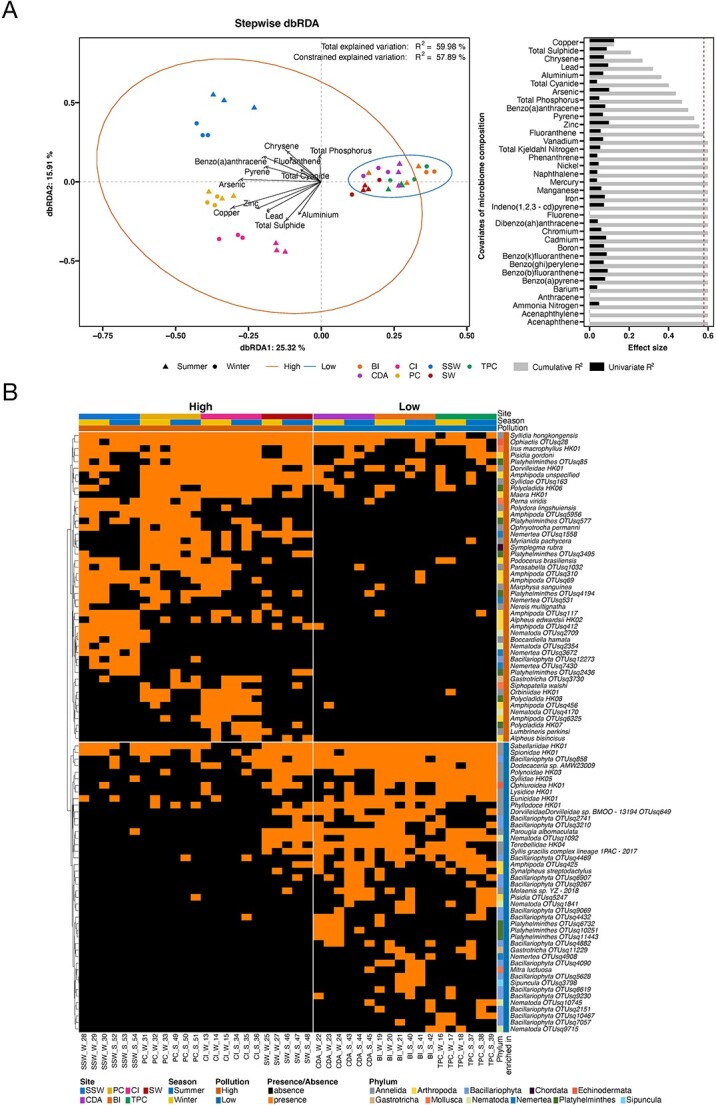
Benthic metazoans affected by pollution. (A) dbRDA plot based on Jaccard distance. Confidence ellipses: 95% confidence level. The dashed line represents the cut-off for significant nonredundant contribution to the multivariate model. (B) Significantly differentially abundant benthic metazoans between low- and high-pollution sites identified by R package MaAsLin2 (*P* ≤ .05, FDR ≤ 0.2).

Next, we compared microbial and pollution data to COI metabarcoding results of all <2 mm motile organisms and homogenized sample of all sessile scrapings from ARMS deployed for 12 months at the seven sites. After quality filtering and keeping only taxa that are known benthic infaunal metazoans (Table S8) [54–62], in total 571 OTUs were included in the downstream analysis (Table S9). Beta diversity of the benthic metazoans assessed using the Jaccard index revealed significantly different communities across the pollution sites at the species level (PERMANOVA, *P* = .001). To understand how benthic metazoans’ biodiversity is related to environmental pollution stress, we constructed a dbRDA with sediment pollution indices. The assemblages formed by high and low-pollution sites was clearly separated. All covariates together explained 60.0% of the benthic metazoan variance at the species level (dbRDA, R^2^ = 60.0%, *P* = .001), which is strikingly similar to the microbiome variance explained by pollution indices. Twelve covariates together explained both significant and nonredundant fractions of variation (Fig. 4A, stepwise dbRDA, R^2^ = 58%, *P* = .001). Thirty-one pollution indices significantly explained the variance of the benthic metazoans in univariate analysis (dbRDA, FDR < 0.05), with heavy metals (copper, arsenic, zinc, and lead), PAH pollutants [Benzo(b)fluoranthene and Benzo(k)fluoranthene] and total sulfide explained the most of variance. Out of all nonredundant covariates, heavy metal pollution indices such as copper, lead, arsenic, and zinc were associated with high-polluted assemblages. Total sulfide, copper, lead, arsenic, pyrene, and total cyanide were found to account for the nonredundant variance in both the microbiome and benthic metazoans.

We identified significantly enriched benthic metazoans for each season using MaAsLin2 (*P* ≤ .05, FDR ≤ 0.2) based on linear mixed-effects models (Fig. S6). More benthic metazoan OTUs were enriched in the summer (35) compared to winter (19), in which OTUs from phyla *Bacillariophyta*, *Gastrotricha*, and *Nematoda* are only enriched in summer. Similar analysis adjusting for season revealed pollution-associated benthic metazoans by R package MaAsLin2 (*P* ≤ .05, FDR ≤ 0.2), including forty-seven pollution-tolerant and forty-four pollution-sensitive OTUs (Fig. 4B). *Bacillariophyta* exhibited the most pollution-sensitive OTUs. Most pollution-associated OTUs belong to the phyla *Annelida*, *Bacillariophyta*, and *Arthropoda*. Specifically, pollution-tolerant OTUs included *Boccardiella hamata* and *Lumbrineris perkinsi* from *Annelida*, as well as *Alpheus bisincisus* and *Alpheus edwardsii* from *Arthropoda*. In contrast, pollution-sensitive OTUs comprised *Parougia albomaculata* from *Annelida*, *Synalpheus streptodactylus* from *Arthropoda*, and *Mitra luctuosa* from *Mollusca*.

### Microbiome mediates the pollution effect on sessile benthic metazoans

**Figure 5:**
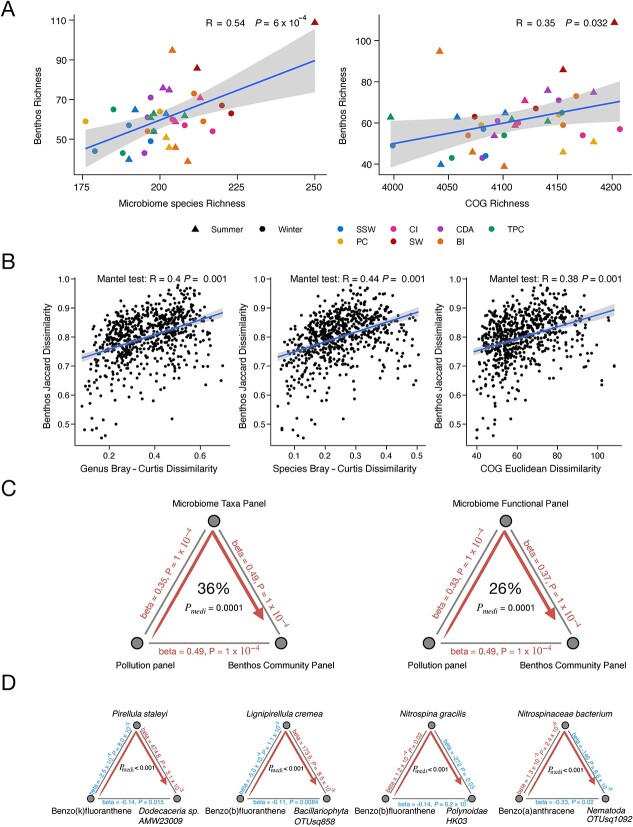
Interactions between microbiome and benthic metazoans under pollution stress. (A) Significant positive associations between microbiome community richness, at both species and function level, and the benthic metazoans richness. (B) Significant associations between the benthic metazoans and microbiome community dissimilarity at genus and species level and functional dissimilarity. (C and D) Mediation linkages illustrating the mediator role of microbial species and microbial functions in the associations between pollution and the benthic metazoans at the level of (C) community and (D) specific microbial species.

By integrating the data from the microbiome and benthic metazoans, we found that the richness of the microbiome taxonomic and functional profiles was significantly positively correlated with the richness (at the OTU level) of benthic metazoans (Fig. 5A, species: R = 0.54, *P* = .0006; function: R = 0.35, *P* = .032). Furthermore, we found statistically positive correlations between the dissimilarity in benthic metazoans and the dissimilarity in bacterial community taxonomic composition and functional composition (Fig. 5B, Mantel test, *P* < .01). To further investigate whether the microbiome community acts as a mediator in the influence of pollution indices on benthic metazoans, we conducted mediation analyses at both the community and individual levels. Mediation analysis is a statistical method that elucidates how an independent variable impacts a dependent variable through an intermediary, known as a mediator. This technique is essential not only for assessing the presence of an effect but also for understanding its underlying mechanism. Notably, mediation analysis with MODIMA showed that microbial communities can mediate the pollution impact on benthic metazoans (Fig. 5C). The panel of microbial species accounted for 36% (*P* < .01) of the total effect of pollution quality on the benthic metazoans, whereas the panel of microbial function accounted for 26% (*P* < .01). We further validated the significant mediation effect (global mediation: *P* < .01) of microbial species and functions with another mediation analysis method LDM-med [43] (Fig. S7).

Subsequently, we performed individual-level mediation analysis based on pollution-associated microbiomes, microbial functions, and benthic metazoans to evaluate the specific microbial species and functions that can mediate the impact of pollution on the benthic metazoans (FDR < 0.05). In total, 142 mediation linkages via microbial species and 20 mediation linkages via microbial functions revealed the effects of pollution on pollution-sensitive benthic metazoans (Tables S10 and S11). Among these, microbes from the phylum *Planctomycetes* were the primary mediators (81 out of 142 mediation linkages). Notably, PAHs exert significant effects on pollution-sensitive phyla such as *Annelida* and *Bacillariophyta* primarily through microbial mediation by *Planctomycetes*, which accounted for 60 linkages. Specifically, our mediation analysis suggested that Benzo(k)fluoranthene and Benzo(b)fluoranthene contribute to a decline of *Dodecaceria_sp. AMW23009* and *Bacillariophyta OTUsq858*, by decreasing *P. staleyi and Lignipirellula cremea* levels, respectively (Fig. 5D). Conversely, *Nitrospinae*, the phylum containing pollution-tolerant microbes, mediated the effects (19 linkages) of pollution on sensitive benthic metazoans including *Annelida* and *Nematoda* (Fig. 5D). From a mechanistic perspective, we identified several microbial functions to mediate the impact of pollution on pollution-sensitive *Annelida*, mainly including Na + −translocating NADH dehydrogenase, FoF1-type ATP synthase, pyrimidine salvage, and molybdopterin biosynthesis (Table S11). Furthermore, we investigated the potential effects of pollution on microbial species mediated by benthic metazoans (Table S12). In total, we identified 102 mediation linkages, 79 of which were mediated by benthic species from the phylum *Annelida*. The mediation effect of microbes was primarily driven by PAH pollution, accounting for 81 out of 142 linkages, while the mediation effect of benthic metazoans was mainly associated with heavy metal pollution, representing 60 out of 102 linkages.

## Discussion

Recent studies have shown that coastal marine microbiomes are impacted by pollutants resulting from fast urbanization and human activities, such as metal contamination, plastic leachates, and organic pollutants [63–65]. Besides the multiple roles attributed to the environmental microbiome, it serves also as a source for the microbiomes associated with metazoans. For instance, the carapace and gut of fiddler crabs are colonized by different pools of microbial colonists acquired from the environment [66], whereas, seawater and marine sediments were identified as microbial sources for the colonization of fish mucus [67]. However, studies examining holistically and in high taxonomic resolution the micro-organisms and benthic metazoans are required to determine how microbial processes influence the ecology of coastal marine ecosystems under pollution stresses. By using geochemical indicators, deep shotgun metagenomics sequencing and COI metabarcoding sequencing, we show that various pollution levels and sources have an effect on microbial populations and functional output, which in turn affect the sessile benthic metazoans.

Overall, our analysis of the shotgun metagenomics data generated using marine sediments under pollution stress suggests that diverse pollutants resulting from human activities in the coastal marine zone of the rapidly developing city impact benthic metazoans by shaping the composition and diversity of marine sediment microbiomes. Redundancy analysis revealed that pollution indices explained >50% of the variance of microbial communities. Our study’s explained variation percentage closely resembles a previous finding that found water environmental stress accounted for ~56% of the variation in the seawater microbiome [68]. Arsenic, total sulfide, ammonia nitrogen, and pyrene are the most influential elements shaping microbial communities. Due to their small size, microbes have a high surface-to-volume ratio and a broad contact area with the environment, which enhances their ability to absorb [69, 70], metabolize, and interact with environmental pollutants [71–74]. At the same time, we observed significant changes in microbial functional pathways under pollutant stress, likely resulting in a unique metabolic landscape that may impact organisms in higher trophic levels. Similar to the redundancy studies based on the microbiome community, >50% of the variance of the benthic metazoans was explained by pollution indices. In addition, we identified both pollution-sensitive and pollution-tolerant microbial species, as well as benthic metazoans, that showed significant changes in abundance in response to increased pollution.

Interestingly, alpha and beta diversity analysis suggested strong positive correlations between the microorganisms and benthic metazoans, whereas mediation analyses revealed a possible role of the microbiome community between pollution and the benthic metazoans. In particular, from the individual-level mediation analysis of pollution-associated features, we identified 142 and 20 linkages through microbes and microbial functions to mediate the pollution impact on the benthic metazoans. We found that microbes from the *Planctomycetes* phylum are involved in more than half of all mediation linkages, significantly outnumbering those mediated by *Proteobacteria*. This is notable given that *Proteobacteria* is the most abundant phylum and contains the highest number of species significantly related to pollution. *Planctomycetes*, as a known host-associated bacteria, often live in association with other eukaryotic organisms, including giant tiger prawn *Penaeus monodon* [75], Algae, and natural sponges [76]. However, the role of *Planctomycetes* in mediating the effects of pollution on benthic metazoans remains largely unexplored. Our study also suggests that microbes from the *Nitrospinae* phylum may reduce the growth of several pollution-sensitive benthic metazoans from the *Annelida* and *Nematoda* phyla under pollution stress. Microbial mechanisms associated with energy synthesis and conservation, such as Na + −translocating NADH dehydrogenase, FoF1-type ATP synthase and pyrimidine salvage [77], play an active role in mediating the impact of pollution on benthic metazoans. Marine microbial communities offer enormous functional resilience [78] and play vital roles in ocean ecology and planet health [79]. Overall, our mediation analyses on chemical pollution indices, microbiome community, and benthic metazoans imply that the compositional and functional changes of the microbiome induced by the pollution might significantly influence the biodiversity and ecological dynamics of benthic metazoans.

However, our study has also limitations. At sites TPC and BI, there is a time difference of up to 2 months between the collection of pollution data and microbial/metazoan samples, which may have introduced minor temporal variability. However, given the persistence of the marine sediment pollution indices [80, 81] we studied, we expect this time difference to have a minimal effect on our results. Additionally, despite a range of pollution indices and environmental variables included in our study, not all possible environmental factors that potentially affect microbiome and benthic metazoans community were considered. A more comprehensive characterization of sediment environmental factors in future research could enable more precise identification of pollution-associated signatures. Our research is primarily correlational and, therefore, does not establish causality, and experimental validation in well-controlled artificial environments is thus needed. An example of an artificial environment is demonstrated in a previous study [82] on benthic annelids in polluted marine sediment. Following the *in vitro* model used for mediation validation in a prior study, the effects of treatments on both the mediator (microbes) and the responder (benthic communities) should first be evaluated independently. To assess the mediation effect of microbes, future experiments should implement sterilized marine sediment [83, 84] and seawater [85] in artificial environments, both with and without microbial presence to examine the influence of microbes on benthic metazoans responses. Furthermore, while it is widely accepted that the sediment microbiome serves as a source for the microbiome of benthic metazoans, characterization of the microbiome of each individual benthic metazoan was not performed in our study.

In summary, integrating pollution indices, the microbiome, and the sessile benthic metazoans has revealed a potential interaction between the bacterial community and benthic metazoans under pollution stress. Most importantly, through our quantitative analysis, we discovered communication channels between the benthic metazoans and microbiome communities in marine sediments under pollution stress. Microorganisms account for the vast majority of the biomass in ocean environments and are accountable for most of the major biogeochemical cycles. Our study demonstrates not only how human activities directly affect the microbiome and benthic metazoans, but also how an altered microbiome composition may affect benthic metazoans in coastal marine environments. These findings also offer novel perspectives on the complex ecological dynamics of coastal marine ecosystems in urbanized coastal metropolises and how could be modified for protecting these fragile ecosystems.

## Supplementary Material

FigS1_ycae141

FigS2_ycae141

FigS3_ycae141

FigS4_ycae141

FigS5_ycae141

FigS6_ycae141

FigS7_ycae141

Supplementary_Tab_S1_ycae141

Supplementary_Tab_S2_ycae141

Supplementary_Tab_S3_ycae141

Supplementary_Tab_S4_ycae141

Supplementary_Tab_S5_ycae141

Supplementary_Tab_S6_ycae141

Supplementary_Tab_S7_ycae141

Supplementary_Tab_S8_ycae141

Supplementary_Tab_S9_ycae141

Supplementary_Tab_S10_ycae141

Supplementary_Tab_S11_ycae141

Supplementary_Tab_S12_ycae141

Xu_Marine_microbiome_Supplementary_Information_ycae141

## Data Availability

The raw Illumina sequence data of metagenomic data have been deposited in the sequence read archive at NCBI under Bioproject (accession ID: PRJNA1035294). All remaining data generated or analyzed during this study are included in this article and its supplementary information files. The scripts for all statistical analyses are available in the following repository: https://github.com/Lin-LinXu/Sediment-microbiome-mediates-the-effect-of-pollution-on-benthic-macro-organisms/.
