# Supplementary figures and images for: Chemical pollution drives taxonomic and functional shifts in marine sediment microbiome, influencing benthic metazoans
Source: ISME Commun. 2025 Feb 13;5(1):ycae141. doi: 10.1093/ismeco/ycae141 (PMC11851482; doi:10.1093/ismeco/ycae141)

a

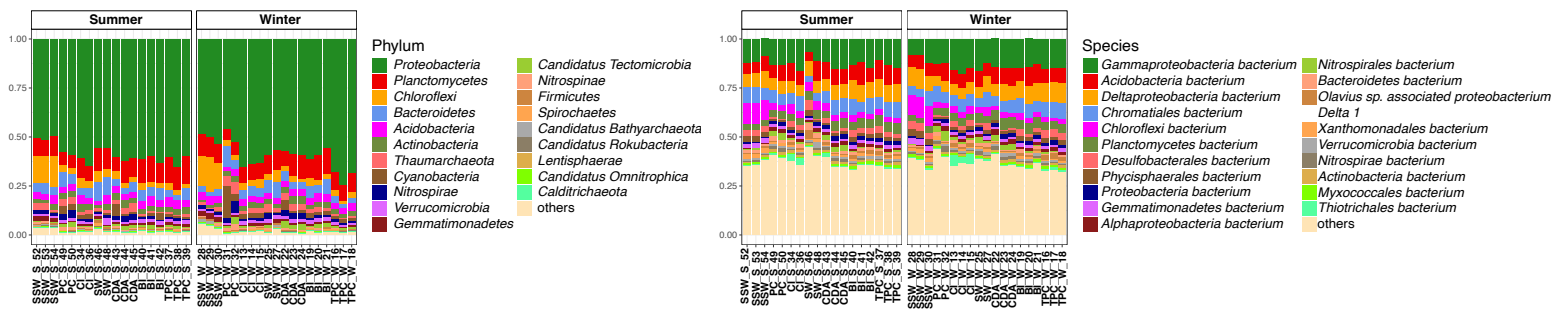

b

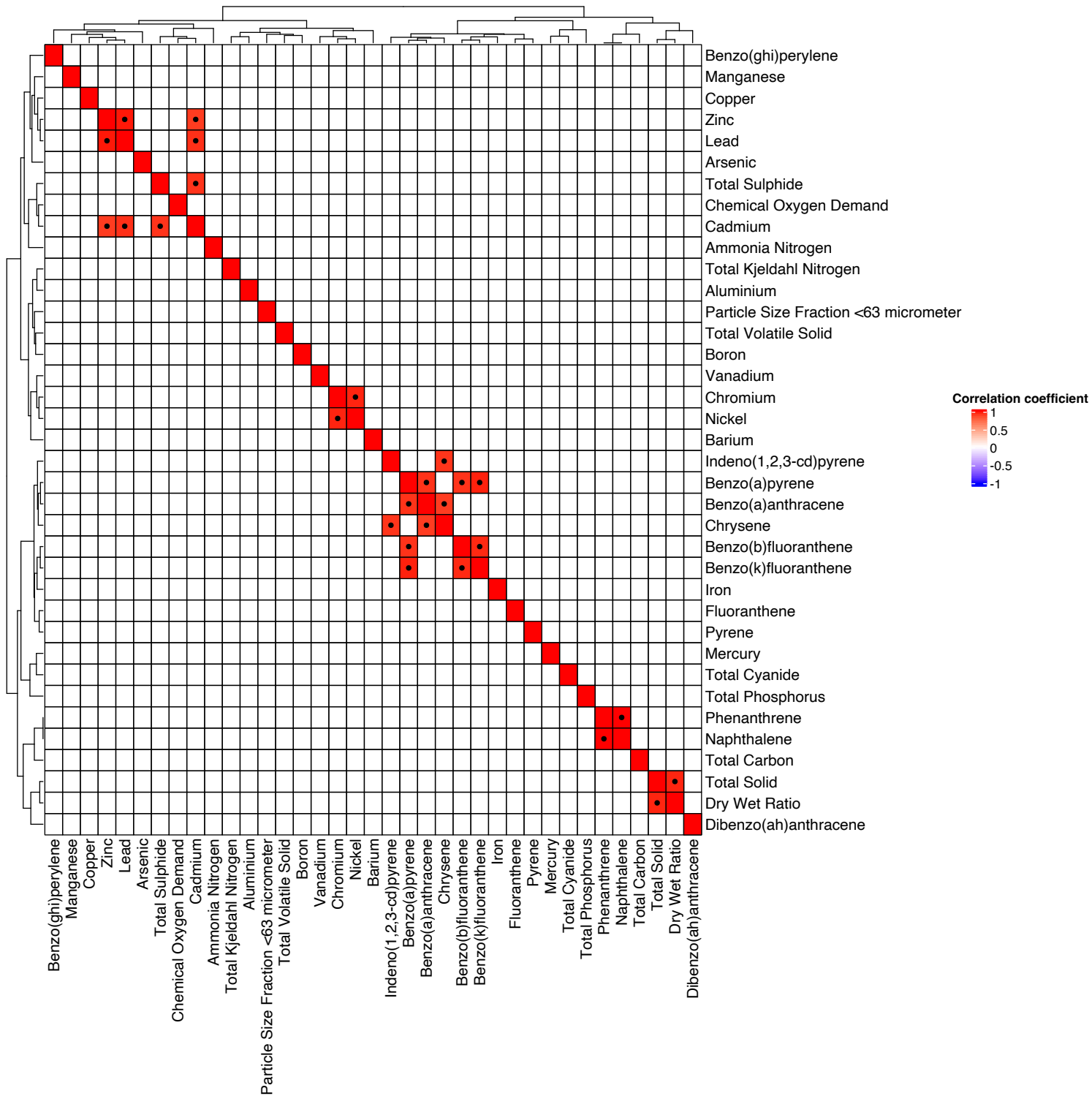

Figure S1

Supplement: FigS1_ycae141 [file figs1_ycae141.pdf]

**a**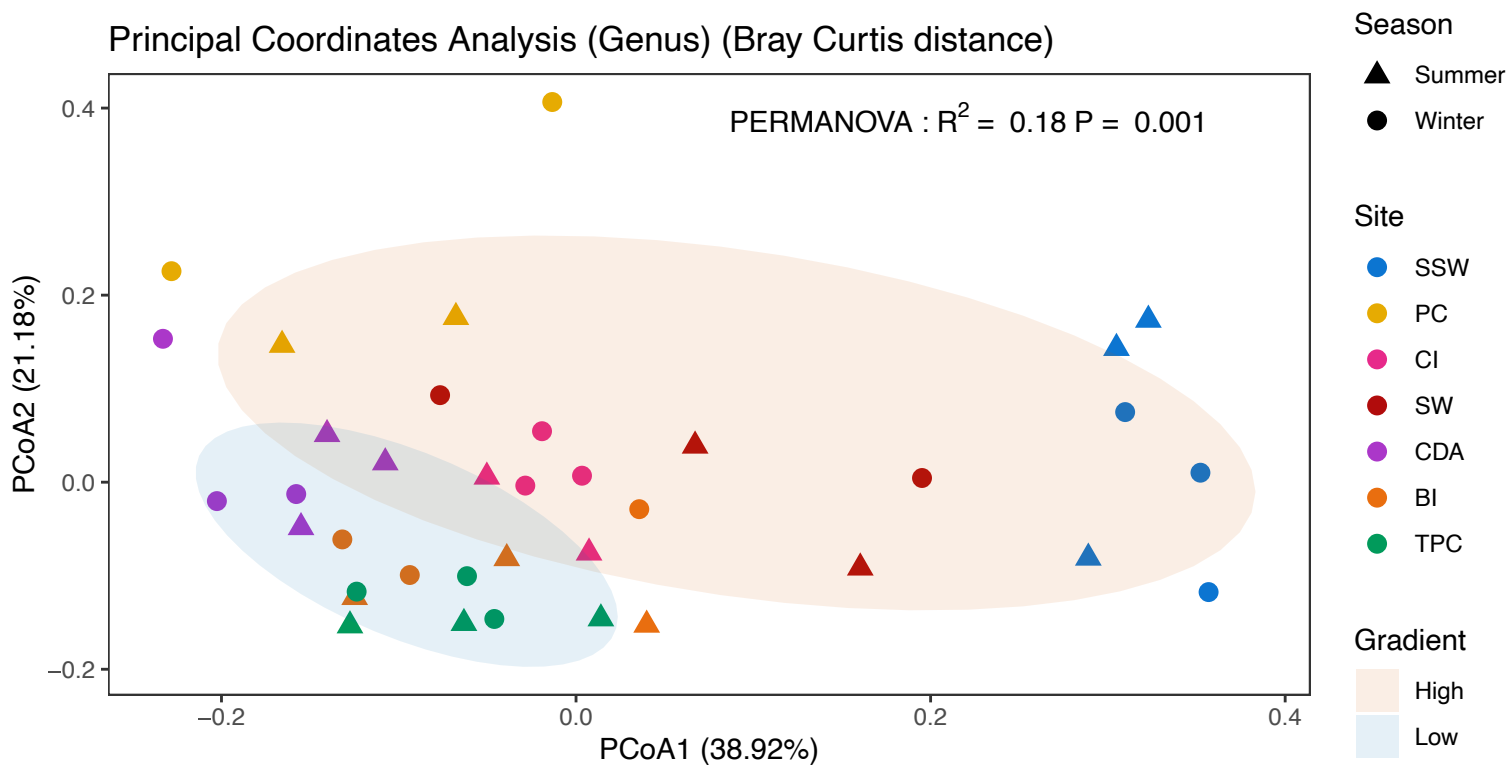**b**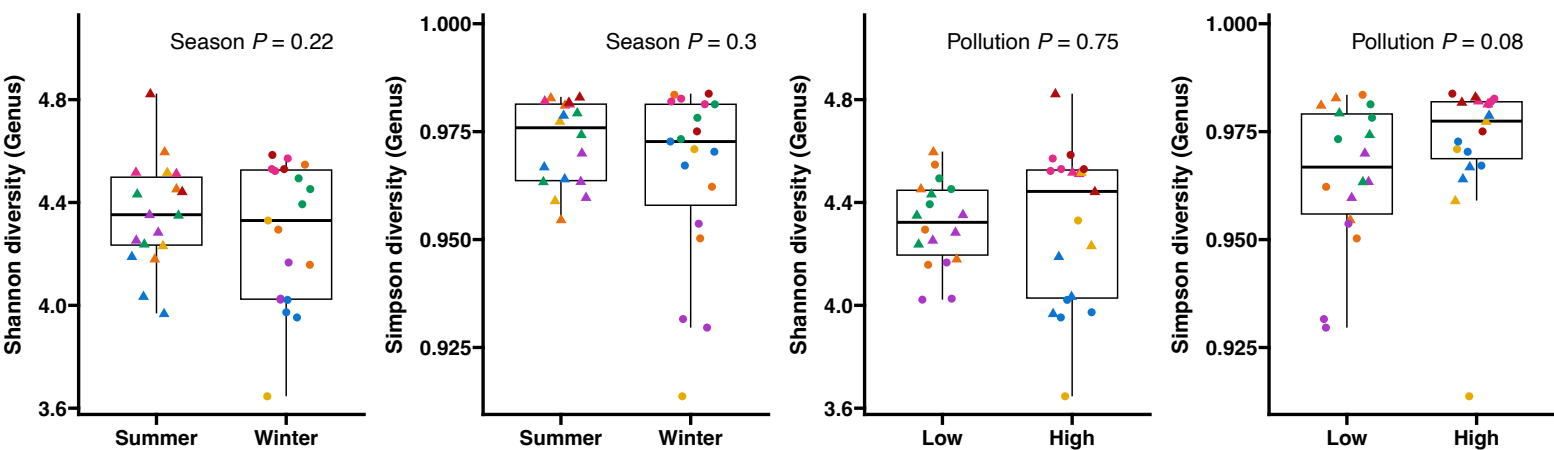**Figure S2**

Supplement: FigS2_ycae141 [file figs2_ycae141.pdf]

a

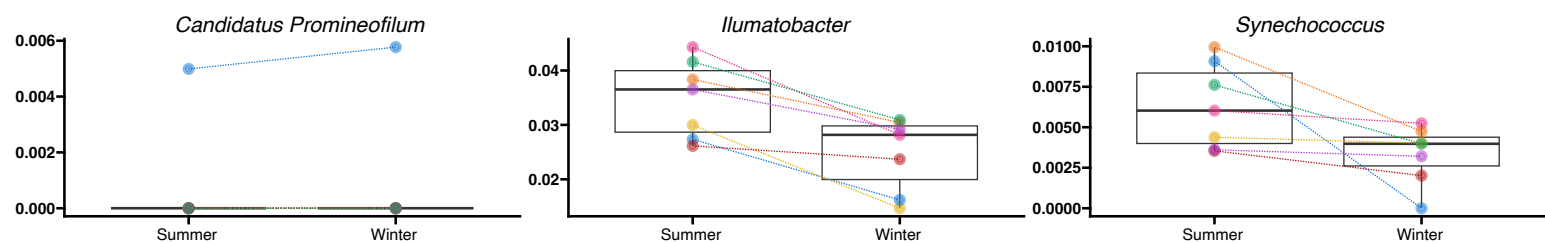

b

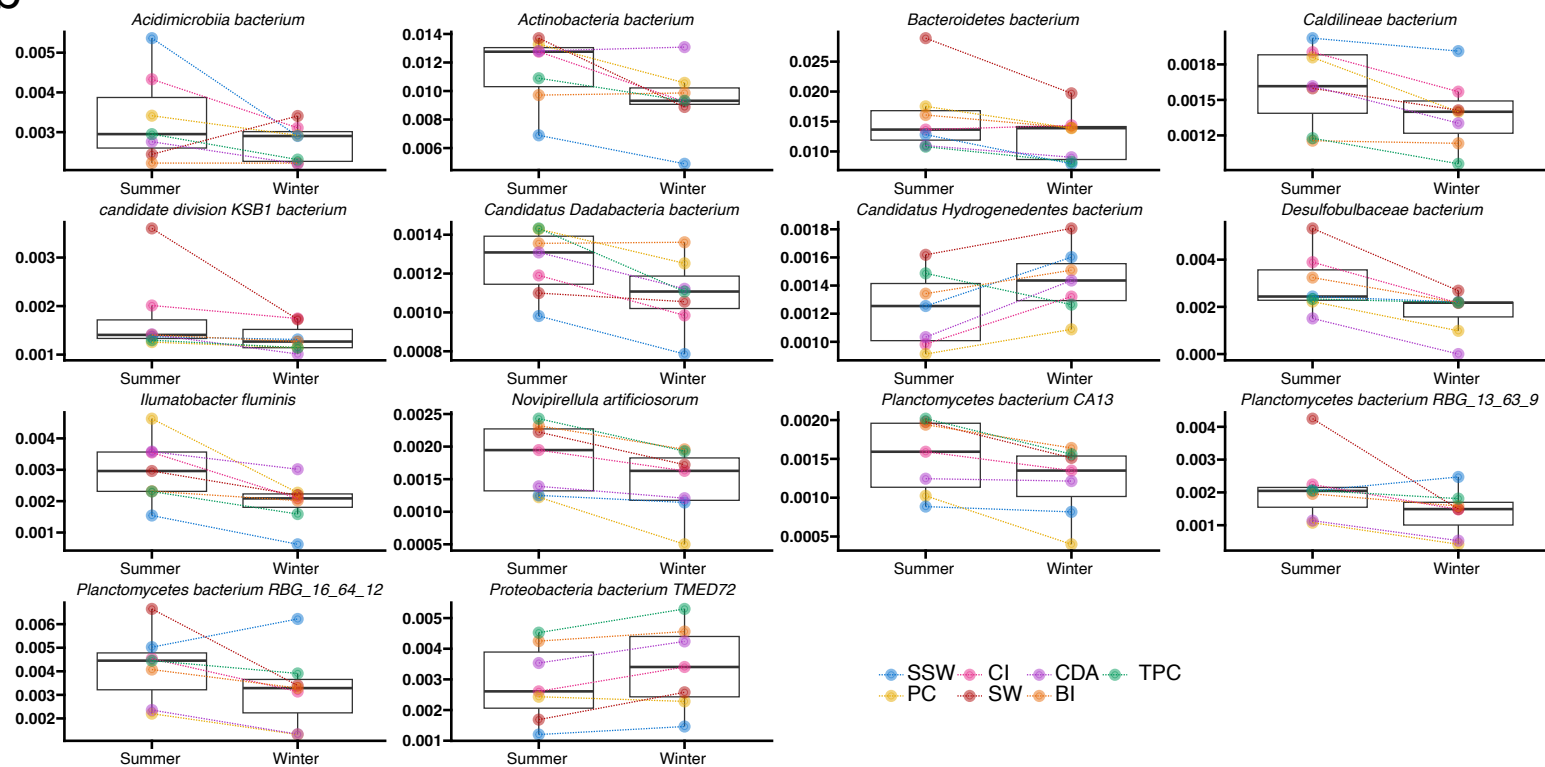

c

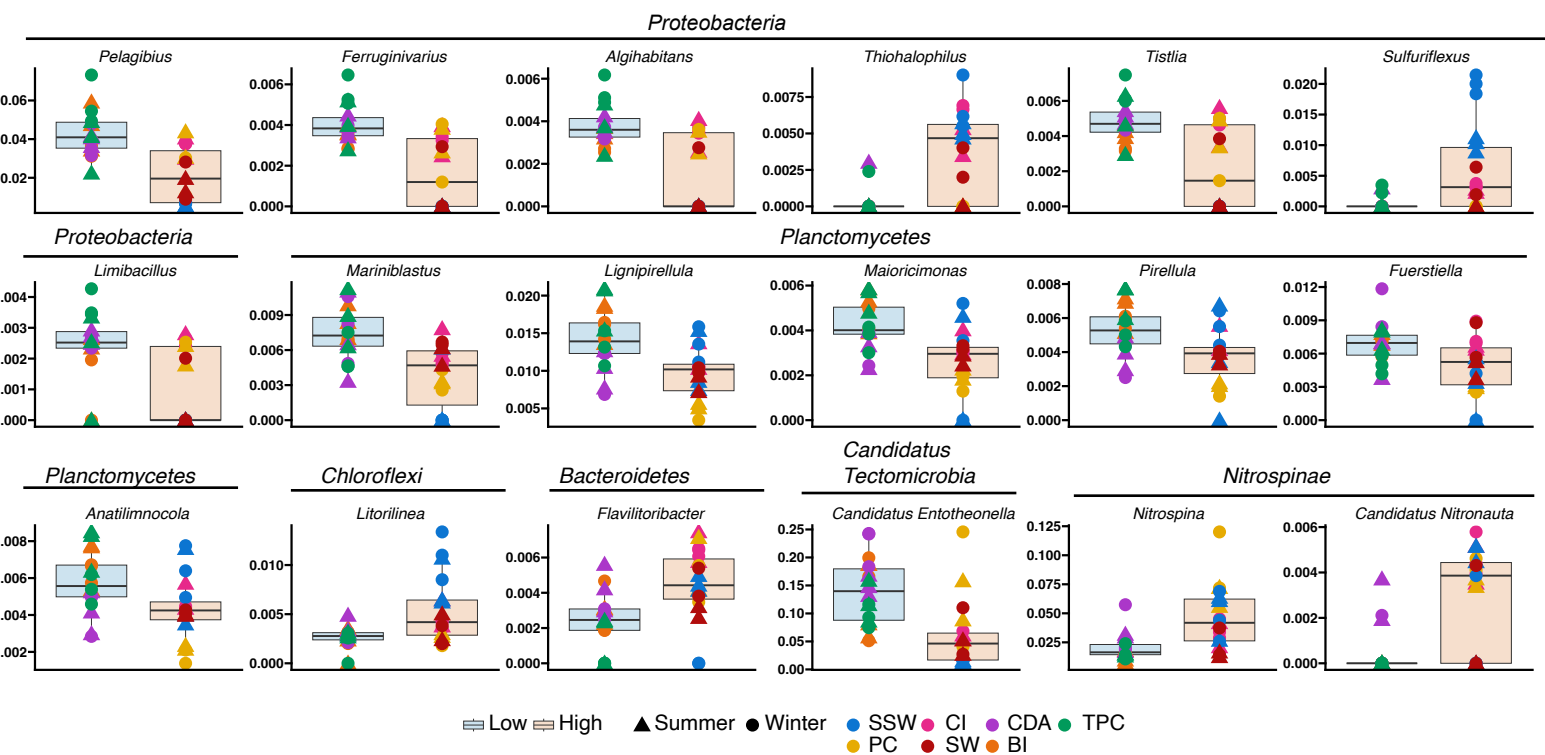

Figure S3

Supplement: FigS3_ycae141 [file figs3_ycae141.pdf]

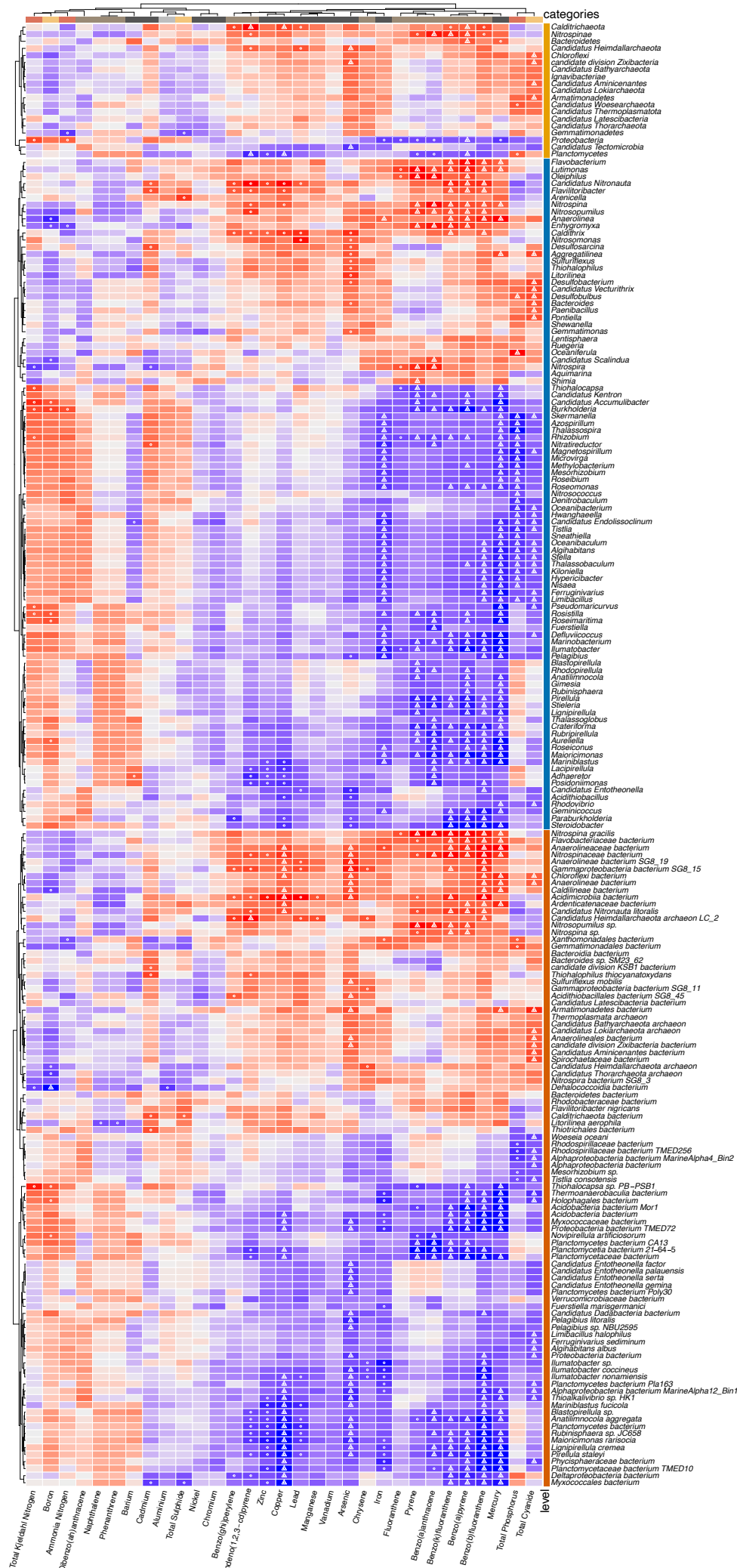

Figure S4

Supplement: FigS4_ycae141 [file figs4_ycae141.pdf]

**a**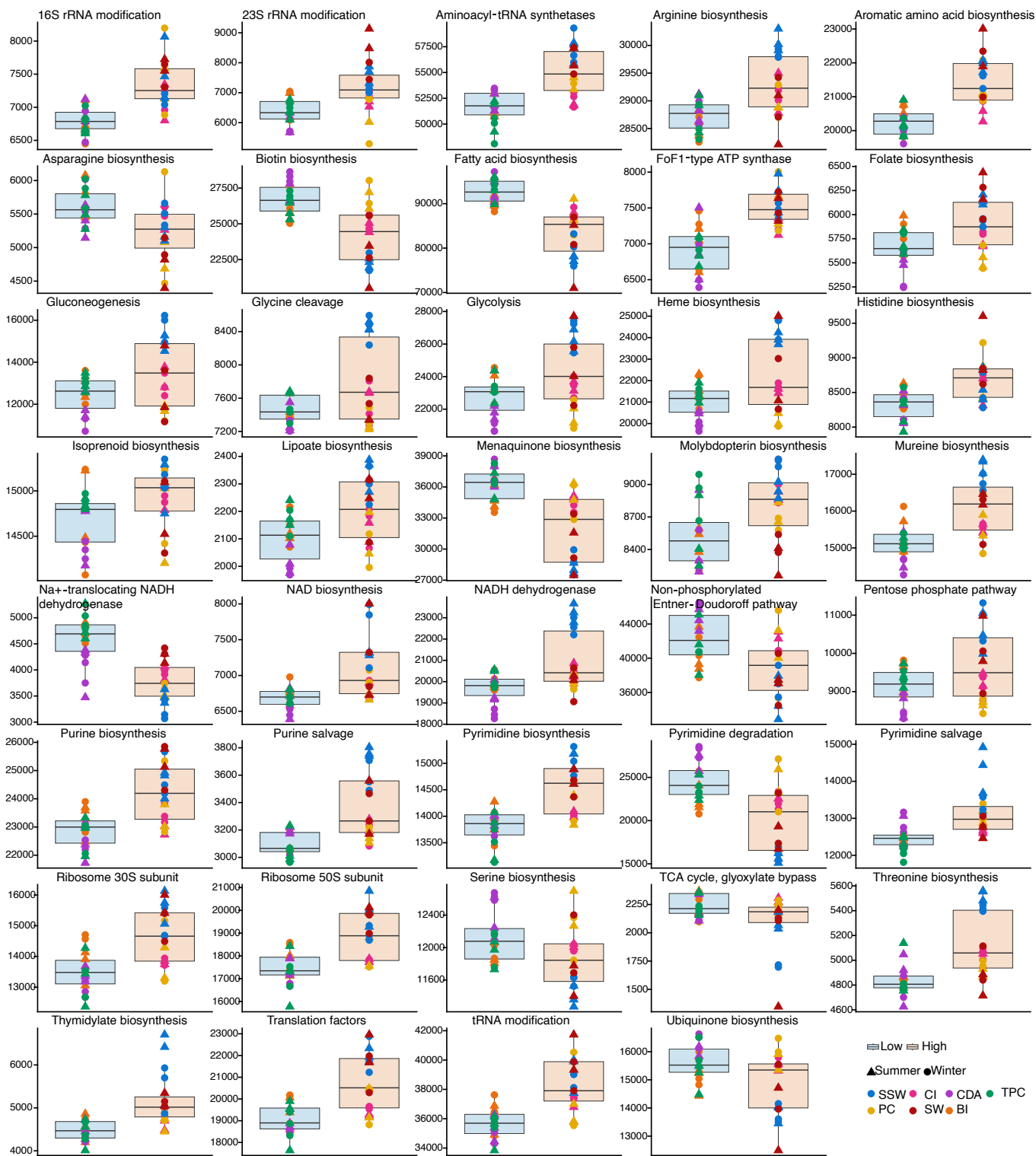**b**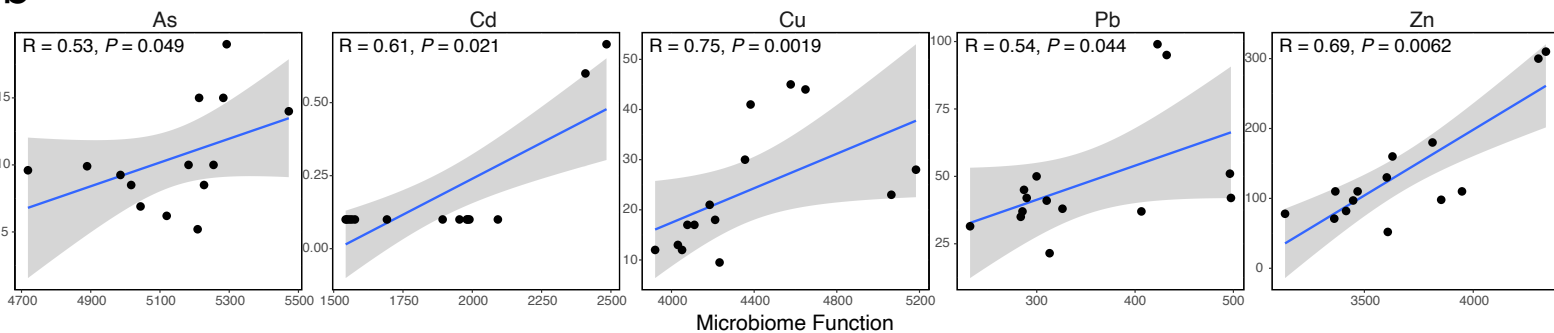**Figure S5**

Supplement: FigS5_ycae141 [file figs5_ycae141.pdf]

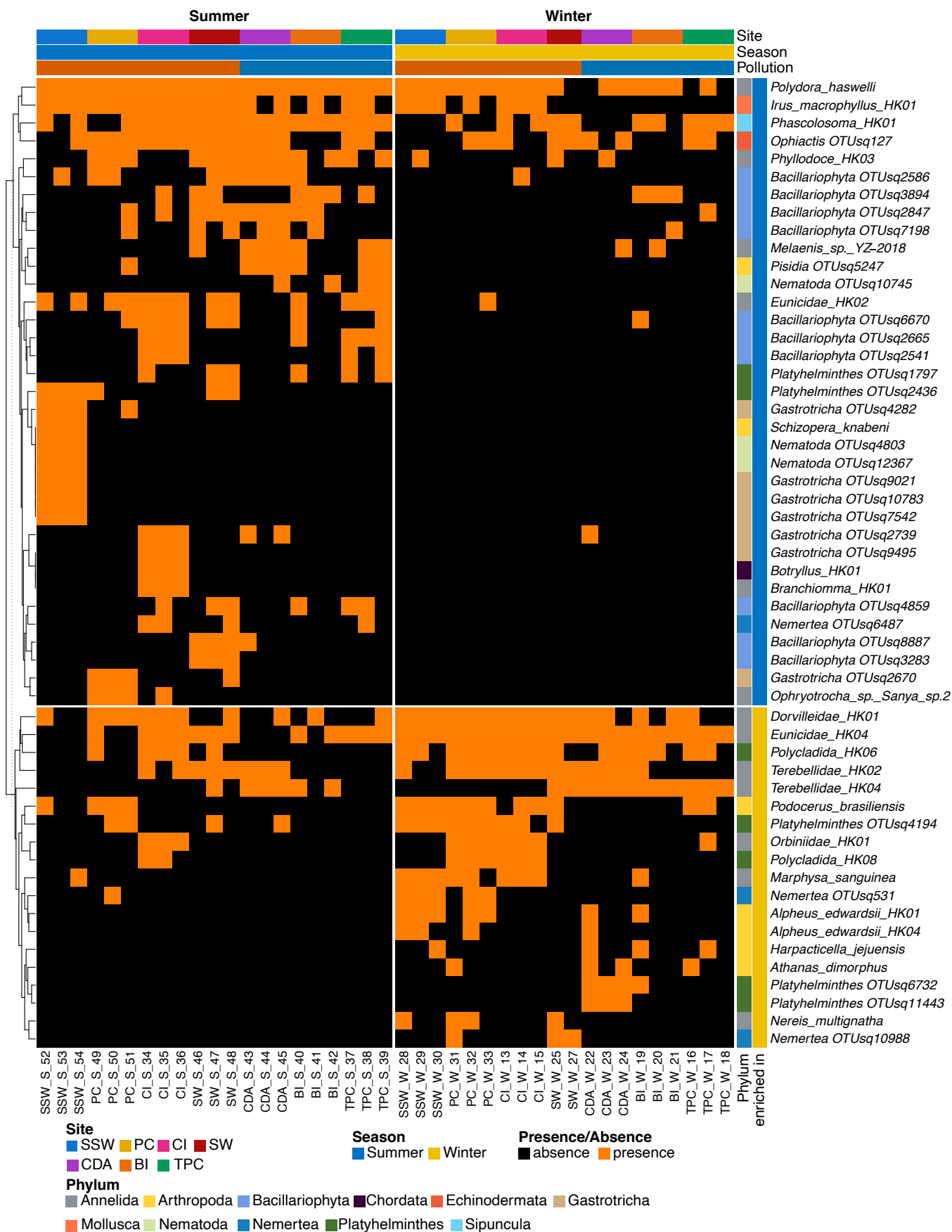

**Figure S6**

Supplement: FigS6_ycae141 [file figs6_ycae141.pdf]

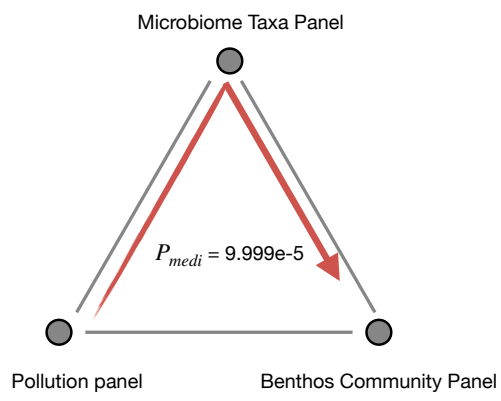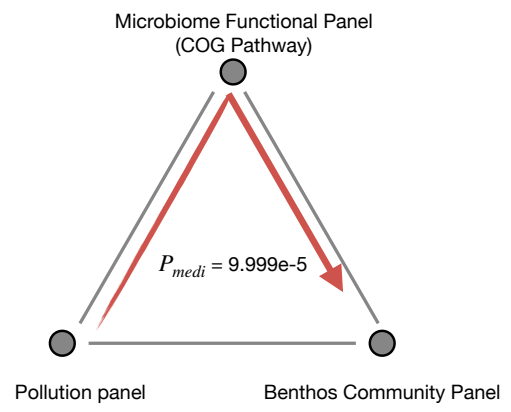

**Figure S7**

Supplement: FigS7_ycae141 [file figs7_ycae141.pdf]
