# Supplementary material for: Chemical pollution drives taxonomic and functional shifts in marine sediment microbiome, influencing benthic metazoans
Source: ISME Commun. 2025 Feb 13;5(1):ycae141. doi: 10.1093/ismeco/ycae141 (PMC11851482; doi:10.1093/ismeco/ycae141)
Supplement: Xu_Marine_microbiome_Supplementary_Information_ycae141 [file xu_marine_microbiome_supplementary_information_ycae141.doc]

**Supplementary Information**

Figure S1: Profiles of the prokaryote community and a multicollinearity correlation plot of pollution indices and environmental variables.

(**a**) Taxonomic profile among samples at phylum and species level with information represented as relative abundance. (**b**) Correlation plot of both pollution indices and environmental variables.

Figure S2: Diversity of the prokaryote community.

(**a**) Beta diversity of prokaryote communities at the genus level. (**b**) Alpha diversity of prokaryote communities.

Figure S3: Microbes affected by pollution and season.

(**a**) Microbes significantly related to season after adjusting for the sampling sites at the genus level. (**b**) Microbes significantly related to season after adjusting for the sampling sites at the species level. (**c**) Microbes significantly related to pollution after adjusting for the season at the genus level.

Figure S4: Microbes corelated with pollution indices.

Correlation between pollution-associated microbes and pollution indices. Correlations with raw P-value < 0.05 were labelled with a circle. Correlations with raw P-value < 0.05 and BH P-value < 0.2 were labelled with a triangle.

Figure S5: Pollution-associated function of the prokaryote community.

(**a**) COG pathways significantly associated to pollution after adjusting for season. (**b**) Heavy metal resistance KOs significantly positively correlated with corresponding heavy metals.

Figure S6: Benthic metazoans affected by season.

Benthic metazoans’ OTUs were significantly associated with season after adjusting for sampling sites using a linear mixed model.

Figure S7: Mediation effect on community level.

Mediation linkages of microbial species and microbial functions (COG pathways) mediate the associations between pollution and the benthic metazoans.

Table S1. Sediment pollution indices and environmental variables in year 2019.

Table S2. Metagenome-seq reads quality.

Table S3. OTU table of prokaryote communities.

Table S4. pollution indices and environmental variables associated with microbiome community (univariate dbRDA).

Table S5. Differential abundance of sediment microbiome associated with pollution.

Table S6. Metagenome-seq assembly quality.

Table S7. Differential abundance of COG terms associated with pollution.

Table S8. Benthic infaunal metazoan taxonomic levels.

Table S9. OTU table of benthic metazoans.

Table S10. Mediation analyses mediated by microbial species.

Table S11. Mediation analyses mediated by microbial functions.

Table S12. Mediation analyses mediated by benthic metazoans.
